# Predicting Breast Cancer Based on Optimized Deep Learning Approach

**DOI:** 10.1155/2022/1820777

**Published:** 2022-03-19

**Authors:** Hager Saleh, Sara F. Abd-el ghany, Hashem Alyami, Wael Alosaimi

**Affiliations:** ^1^Faculty of Computers and Artificial Intelligence, South Valley University, Hurghada, Egypt; ^2^Faculty of Computing and Information, Luxor University, Luxor, Egypt; ^3^Department of Computer Science, College of Computers and Information Technology, Taif University, P. O. Box 11099, Taif 21944, Saudi Arabia; ^4^Department of Information Technology, College of Computers and Information Technology, Taif University, P. O. Box 11099, Taif 21944, Saudi Arabia

## Abstract

Breast cancer is a dangerous disease with a high morbidity and mortality rate. One of the most important aspects in breast cancer treatment is getting an accurate diagnosis. Machine-learning (ML) and deep learning techniques can help doctors in making diagnosis decisions. This paper proposed the optimized deep recurrent neural network (RNN) model based on RNN and the Keras–Tuner optimization technique for breast cancer diagnosis. The optimized deep RNN consists of the input layer, five hidden layers, five dropout layers, and the output layer. In each hidden layer, we optimized the number of neurons and rate values of the dropout layer. Three feature-selection methods have been used to select the most important features from the database. Five regular ML models, namely decision tree (DT), support vector machine (SVM), random forest (RF), naive Bayes (NB), and *K*-nearest neighbor algorithm (KNN) were compared with the optimized deep RNN. The regular ML models and the optimized deep RNN have been applied the selected features. The results showed that the optimized deep RNN with the selected features by univariate has achieved the highest performance for CV and the testing results compared to the other models.

## 1. Introduction

Breast cancer (BC) is one of the most frequent malignant tumors in the world, accounting for 10.4% of all cancer deaths in women aged between 20 and 50 [1]. According to the World Health Organization figures, 2.3 million women will be diagnosed with BC in 2020. BC has been diagnosed in 7.8 million women in the previous 5 years, making it the most frequent malignancy worldwide. BC causes more disability-adjusted life years (DALYs) in women worldwide than any other type of cancer. BC strikes women at any age after puberty in every country on the planet, with rates rising as they become older. For all of these reasons, there is an ongoing need for a reliable and accurate system that can be used to help in the early detection and diagnosis of BC diseases to reduce the number of deaths. In the field of medical analysis, machine-learning (ML) algorithms can be applied extensively [2], for example, predicting COVID-19 [3], predicting Alzheimer's progression[4], predicting chronic diseases [5], predicting liver disorders [6], heart disease [7], cancer [8], and others [9, 10]. ML and deep learning (DL) play a significant role in solving health problems and identifying diseases, such as cancer prediction. Many researchers have applied ML and DL techniques to develop models and systems to predict BC. For example, Asri et al. [11] applied ML algorithms, namely, decision tree (DT), support vector machine (SVM), naive Bayes (NB), and *K*-nearest neighbor (KNN) algorithm on the Breast Cancer Wisconsin (Diagnostic) Data set (BCWD) to predict BC. The result indicated that the SVM classifier was the best. Naji et al. [12] applied five ML algorithms: SVM, random forest (RF), logistic regression (LR), DT, and KNN on BCWD to predict BC. The results demonstrated that SVM had registered the highest accuracy. On their part, Amrane et al. [13] applied ML algorithms, KNN, and NB on BCWD database to predict BC. The results showed that KNN achieved the highest accuracy. Bayrak et al. [14] have applied SVM and artificial neural network on BCWD to predict BC. The results revealed that the best model is registered by SVM. Islam et al. [15] applied five ML techniques: SVM, KNN, RF, LR, and ANNs on BCWD to predict BC. The results showed that the ANNs registered the highest performance. Abdel-Zaher et al. [16] proposed deep neural networks (DNNs) that consist of three hidden layers and two dropout layers to predict BC. They used the BCWD to make the experiment. The results proved that DNN model has achieved the best performance. Also, Prananda et al. [17] proposed a DNN model that consists of three hidden layers and two dropout layers to classify BC. They applied DNN model, SVM, NB, and RF on BCWD data set. The results revealed that the DDN model has registered a significant performance. Soliman et al. [18] designed a hybrid approach based on DNN to improve the classification accuracy. Karaci [19] proposed a DNN model with four hidden layers and two output layers that classify women with or without breast cancer. Nahid et al. [20] proposed the DNN technique for breast cancer picture classification using convolutional neural networks (CNNs), long short-term memory (LSTM), and a combination of CNN and LSTM. Also, Darapurredy et al. [21] used the deep neural network for classifying breast cancer.Feature-selection methods are used to reduce the number of features and selected the subset of features that improve the performance of classification algorithms. For example, Habib et al. [22] applied the genetic programming (GP) as the feature-selection method to select the important features from the BCWD database. They applied nine ML algorithms, namely SVM, KNN, RF, LR, DT, NB, gradient boosting (GB) classifier, AdaBoost (AB) classifier, and linear discriminant analysis (LDA) to select features to predict BC. The results showed that LR, LDA, and GNB algorithms fit best compared to the other methods. Luo et al. [23] used two feature-selection methods: forward selection (FS) and backward selection (BS) to reduce the number of features and improve accuracy. They applied SVM, DT, and ensemble techniques on the BCWD database to predict BC. The results indicated that the ensemble technique with the feature-selection methods had achieved the best performance. Emina et al. [24] used GA feature-selection methods to select the best subfeatures from the BCWD database. They applied different algorithms, namely RF, LR, DT, SVM, DT, and multilayer perceptron (MLP) to select the features, the full features, and the ensemble techniques on BCWD to predict BC. The results showed that RF with GA had recorded the highest performance.This study used feature-selection methods, ML algorithms, DL algorithms, and optimization methods to predict BC. The main contribution is to propose an optimized deep RNN model to predict BC and enhance the results based on recurrent neural networks (RNNs) and the Keras–Tuner optimization technique. Three feature-selection approaches have been employed to select the essential features from the database. The optimized deep RNN is compared to five regular ML algorithms: DT, SVM, RF, NB, and KNN.The remainder of the paper is structured as follows.  Section 2 describes the proposed models and methodologies of predicting BC.  Section 3 presents the experimental results of using the proposed model. Finally, section 5 concludes the paper.

## 2. Methodology

**Figure 1:**
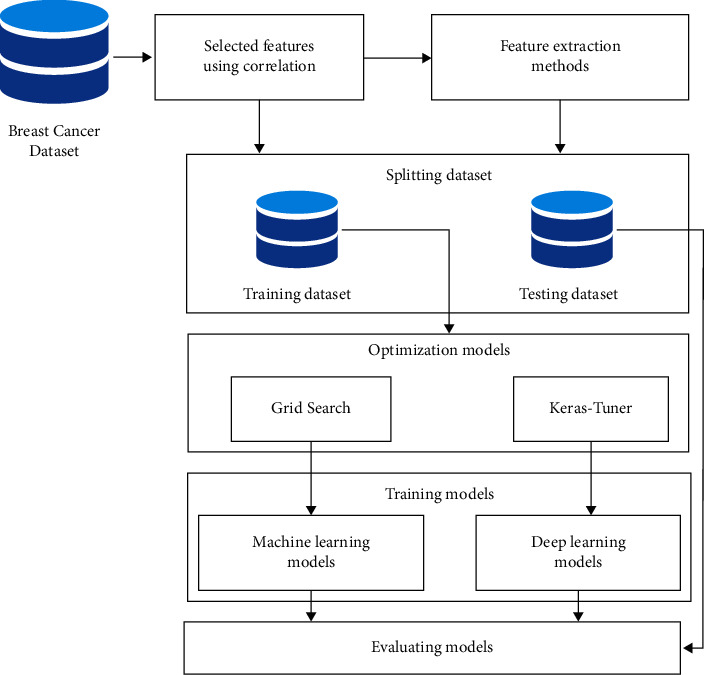
The main steps of the proposed system of predicting BC.

The proposed system of predicting BC consists of two approaches: regular machine-learning (ML) approach and deep learning (DL) approach. In regular ML approach, five ML models are used, namely DT, SVM, RF, NB, and KNN to train and evaluate the BCWD data set. Grid search with cross-validation is used to optimize ML algorithms. In the DL approach, an optimized deep RNN model is proposed and optimized using Keras–Tuner optimization technique. The steps of the proposed system include feature-selection method, spitting database, optimization and training the models, and evaluating the models as shown in Figure 1.

### 2.1. Breast Cancer Data set

We used Breast Cancer Wisconsin (Diagnostic) Data set (BCWD) to train and evaluate the models [25]. The data set includes 30 features and one class label. These features describe the cell nuclei detected in the breast picture clip. The class label has two possible values: 0 or 1. Breast cancer can be classified as benign or malignant, with 0 indicating benign and 1 indicating malignant. The description of features is presented in Table 1.

### 2.2. Feature-Selection Methods

The key advantages of employing feature-selection algorithms are that they allow us to identify the most essential features in a data set.

We used correlation to reduce the number of features in this study and then applied two types of feature-selection algorithms to the data that remained after correlation: univariate feature selection and recursive feature elimination (RFE).

- Correlation methods: we studied the correlation between features using a correlation matrix [26]. We removed one of the features that have a strong correlation with other features of greater than 90%. We chose 17 features from the database after applying the correlation.
- Univariate feature selection works by selecting the best features based on univariate statistical tests. It assigns scores for each feature and the best features that have the highest score [27].
- Recursive feature elimination (RFE) is a wrapper-type feature-selection algorithm. RFE assigned scores for each features, and features that have the highest scores will be extracted. Scikit-learn library [28] is used to apply RFE with random forest.

### 2.3. Splitting Data Set

The BCWD data set is divided into two parts: a training set and a testing set. We employed stratified CV to train and optimize the models with the training set, and the results of CV were recorded for each model. Models are evaluated using a testing set, and the results of the testing set were recorded for each model.

### 2.4. Models Optimization and Training

#### 2.4.1. Regular ML Approach

In regular ML approach, five ML algorithms, such as decision tree (DT) [29], support vector machine (SVM) [30], *K*-nearest neighbor algorithm (KNN) [31], random forest (RF) [32], and naive Bayes (NB) [33] were used to compare with the optimized deep RNN. Grid search with cross-validation is used to optimize ML algorithms and improve ML algorithms performance. Grid-search is used to determine the best hyper-parameters for ML algorithms in order to get the best results. Grid search specifies a set of values for each parameter and then tests each value and chooses the best values for the parameters that yield the best results. CV separates the data set into *k* subsets in order to train ML algorithms on *k*−1 subsets (the training set). The remainder is used to test ML algorithms [29].

#### 2.4.2. Deep Learning Approach

**Figure 2:**
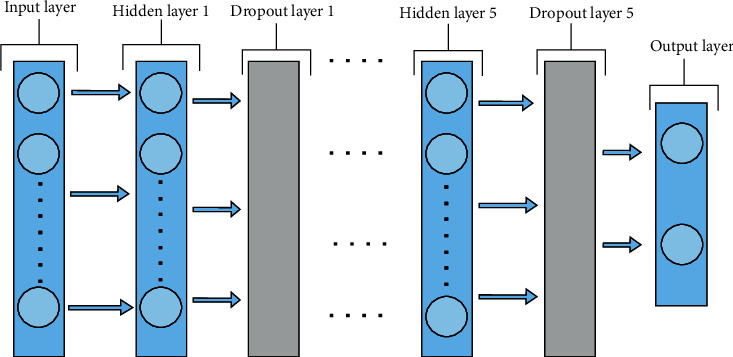
The architecture of the optimized deep RNN model.

We proposed an optimized deep RNN model for breast cancer diagnosis based on recurrent neural networks (RNNs) and the Keras–Tuner optimization technique.  Figure 2c displays the architecture of the optimized deep RNN model that consists of input layer, five hidden layers, five dropout layers, and one output layer. The input layer consists of the number of neurons, input_dim that equals the number of features, kernel_initializer is he_uniform and the activation function is relu. Each hidden layer consists of the number of neurons, the activation function is relu and kernel initializes the_uniform [34]. The output layer consist of two neurons, sigmoid is the activation function and kernel initializes is glorot_uniform. The Keras–Tuner optimization technique [35] is used to optimize the deep RNN model. It is a scalable, easy-to-use hyperparameter optimization system that alleviates the problems associated with hyperparameter search. With a define-by-run syntax, you can easily build your search space and use one of the available search algorithms to identify the optimum hyperparameter values for your models. Keras–Tuner optimization technique has built-in Bayesian optimization, hyperband, and random search algorithms, as well as the ability for researchers to enhance it to try out new search methods.  Table 2 presents the values of the hyperparameters that have been adapted for the optimized deep RNN. Dropout has been applied to hidden layers with the probability of retaining from 0.1 to 0.9. The number of neurons have adapted from 50 neurons to 700 neurons.

### 2.5. Evaluating Models

As illustrated in equations (1) to (4), the models are evaluated using four methods: accuracy (AC), precision (PR), recall (RE), and *F*-measure (FM), where TP indicates true positive, TN indicates true negative, FP indicates false positive, and FN indicates false negative.

(1) $$\begin{array}{c} \text{AC}=\frac{\text{TP}+\text{TN}}{\text{TP}+\text{FP}+\text{TN}+\text{FN}}\text{,} \end{array}$$

(2) $$\begin{array}{c} \text{PR}=\frac{\text{TP}}{\text{TP }+\text{ FP}}\text{,} \end{array}$$

(3) $$\begin{array}{c} \text{RE}=\frac{\text{TP}}{\text{TP}+\text{FN}}\text{,} \end{array}$$

(4) $$\begin{array}{c} \text{FM}=\frac{2\cdot \text{precision}\cdot \text{recall}}{\text{Precision}+\text{recall}}. \end{array}$$

## 3. Experiments and Results

### 3.1. Experiment Setup

This paper's experiments were run on Python 3 and a GPU. The optimized deep RNN was implemented using the Keras package. The ML models were implemented using the scikit-learn package. The data set was divided into two parts: an 80% training set for optimizing the models and registering cross-validation (CV) results and a 20% testing data set (unseen data) for evaluating the models and registering the testing results. First, we studied the correlation between features and removed features that have high correlation above 90% with other features. After that we applied two feature-selection methods on the selected features by correlation to select eight features. Next, the regular ML models and the optimized deep RNN models were applied to the selected features by correlation, selected features by univariate, and selected features by RFE. We adapted some parameters of the optimized deep RNN for each experimental batch size = 10 and epochs = 100. All of the trials were repeated four times in total. The results of CV and the testing of each experiment will be discussed in detail.

### 3.2. Results of Studying Correlation between Features, and ML, and DL Approaches

**Figure 3:**
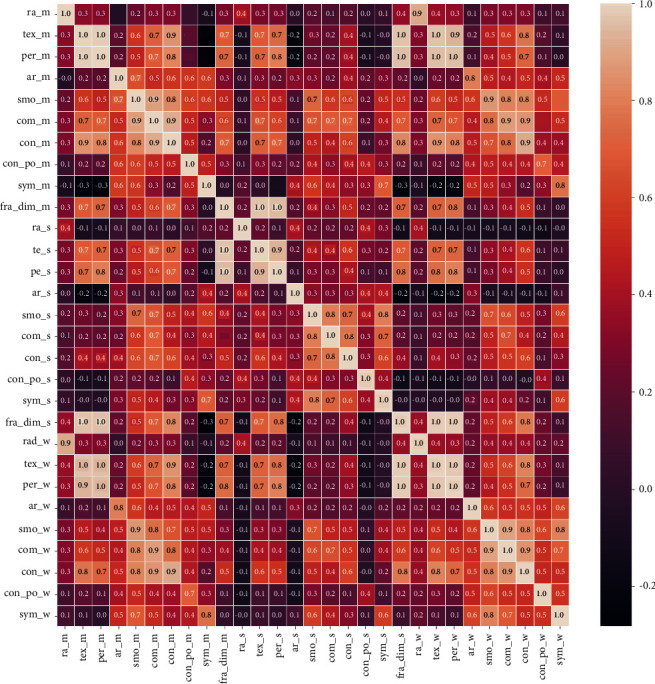
Correlation matrix for breast cancer data set.

As seen in heat map Figure 3, ra_m, per_m, and ar_m are correlated, so ar_m is selected. Com_m, con_m, and con_po_m are correlated with each other. Therefore con_m is selected. Apart from these, ra_se, per_s, and ar_s are correlated, so ar_s is selected. Ra_w, per_w, and ar_w are correlated, so ar_w is selected. Com_w, con_w, and concave po_w are correlated, so con_w is selected. Com_s, con_s, and con_po_s, are correlated, so con_s is selected. tex_m and tex_wo are correlated, and tex_m is selected. ar_w and ar_m are correlated, so ar_m is selected. The final results of the selected features is 16 features.

The results of applying ML models and the proposed model to the selected features by correlation are shown in Table 3. The results of CV performance and testing performance will be described in two subsections.

#### 3.2.1. The Performance of CV Results

In ML approach, the highest performance is registered by RF (AC = 97.01%, PR = 96.74%, RE = 96.75%, and FM = 96.68%), while the worst performance is registered by NB (AC = 81.84%, PR = 82.38%, RE = 81.84%, and FM = 81.01%). The second-highest performance is recorded by SVM (AC = 94.73%, PR = 94.94%, RE = 94.73%, and FM = 94.66%). In DL approach, the optimized deep RNN has enhanced AC by 0.91%, PR by 1.03%, RE by 1.04%, and FM by 1.1%.

#### 3.2.2. The Performance of the Testing Results

In ML approach, the highest performance is registered by LR (AC = 94.04%, PR = 94.05%, RE = 94.04%, and FM = 94.03%), while the worst performance is registered by NB (AC = 83.68%, PR = 84.33%, RE = 84.33%, and FM = 83.0%). The second-highest performance is recorded by SVM (AC = 93.86%, PR = 93.85%, RE = 93.86%, and FM = 93.84%). In DL approach, the optimized deep RNN has enhanced AC by 1.14%, PR by 1.39%, RE by 1.14%, and FM by 1.18%.

Table 4 shows the number of neurons and dropout value in each layer for the optimized deep RNN that is applied on selected features by correlation matrix.

### 3.3. Results of Univariate Feature-Selection Method and ML and DL Approaches

After selecting 17 features of applying correlation matrix, the univariate feature-selection method is applied to 17 features, and 11 features that have the highest scores will be selected. The scores of all features of applying univariate to 17 features are shown in Table 5. We can see that ar_m has the highest score at 53,991.65592, which is the most important feature for breast cancer diagnosis, while fr_di_m has the lowest score at 7.43E−05. We selected 11 features that have the highest score: area_m, ar_s, tex_m, con_w, con_m, sym_w, con_s, smo_w, sym_m, fra_dim_w, and smo_m.

The results of applying ML models and the proposed model to select features by univariate are shown in Table 6. The results of CV performance and the testing performance will be described in two subsections.

#### 3.3.1. The Performance of CV Results

In ML approach, the highest performance is registered by RF (AC = 96.57%, PR = 96.52%, RE = 96.44%, and FM = 96.41%), while the worst performance is registered by NB (AC = 80.74%, PR = 81.22%, RE = 80.74%, and FM = 79.85%). The second-highest performance is recorded by DT RF (AC = 95.17%, PR = 96.52%, RE = 96.44%, and FM = 96.41%). In DL approach, the optimized deep RNN has enhanced AC by 3.32%, PR by 3.37, RE by 3.45%, and FM by 3.48% rather than ML approach.

#### 3.3.2. The Performance of the Testing Results

For the testing result, the highest performance is registered by RF (AC = 94.00%, PR = 94.00%, RE = 94.00%, and FM = 94.00%), while the worst performance is registered by NB (AC = 83.51%, PR = 84.09%, RE = 83.51%, and FM = 82.84%). The second-highest performance is recorded by SVM (AC = 93.86%, PR = 93.85%, RE = 93.86%, and FM = 93.84%). In DL approach, the optimized deep RNN has enhanced AC by 2.74%, PR by 2.39, RE by 2.74%, and FM by 2.8% rather than ML approach.

Table 7 shows the number of neurons and dropout value in each layer for the optimized deep RNN that is applied on the selected features by univariate.

### 3.4. Results of RFE Feature-Selection Method and ML and DL Approaches

**Figure 4:**
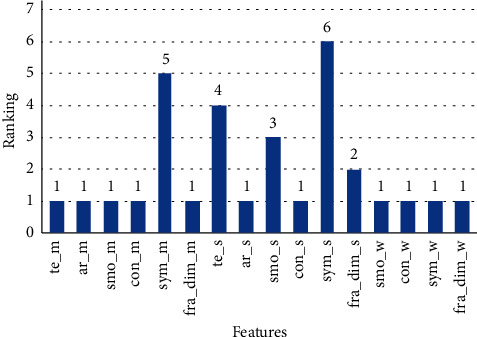
The ranking of features of applying REF.

RFE algorithm sets some of the rankings for each feature. We applied REF to 16 features after coloration and selected the 11 features which ranked the best. The ranking of features is shown in Figure 4. te_m, a_m, smo_m, con_m, ar_s, con_s', fra_dim_s, smo_w, con_w, sym_w, and fra_dim_w have ranked the best, while sym_s and sym_m have registered the worst ranking as 5 and 6, respectively.

The results of applying ML models and the proposed model to the selected features by RFE are shown in Table 8. The results of CV performance and the testing performance will be described in two subsections.

#### 3.4.1. The Performance of CV Results

In ML approach, the highest performance is registered by RF (AC = 96.57%, PR = 96.72%, RE = 96.48%, and FM = 96.45%), while the worst performance is registered by NB (AC = 80.74%, PR = 81.22%, RE = 80.74%, and FM = 79.85%). The second-highest performance is recorded by DT (AC = 94.24%, PR = 94.48%, RE = 94.24%, and FM = 94.4%). In DL approach, the optimized deep RNN has enhanced AC by 1.35%, PR by 1.05, RE by 1.31%, and FM by 1.33%.

#### 3.4.2. The Performance of the Testing Results

In ML approach, the highest performance is registered by RF and SVM (AC = 93.86%, PR = 93.86%, RE = 93.86%, and FM = 93.86%) and (AC = 93.86%, PR = 93.85%, RE = 93.86%, and FM = 93.84%), respectively, while the worst performance is registered by NB (AC = 83.51%, PR = 84.09%, RE = 83.51%, and FM = 82.84%). In DL approach, the optimized deep RNN has enhanced AC by 1.32%, PR by 1.58, RE by 1.32%, and FM by 1.35%.

Table 9 shows the number of neurons and dropout value in each layer for the optimized deep RNN that is applied on the selected features by REF.

## 4. Discussion

**Figure 5:**
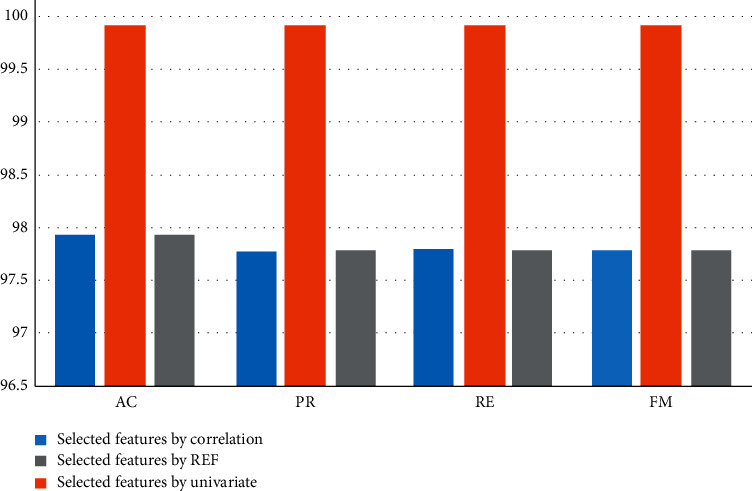
CV results for the optimized deep RNN.

**Figure 6:**
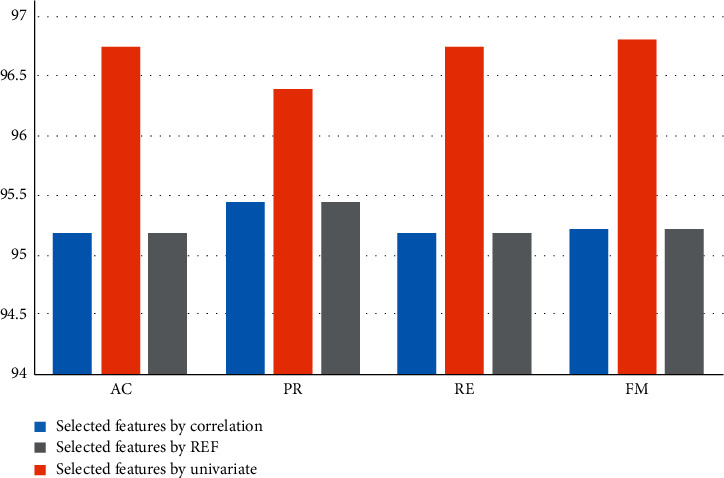
The testing results for the optimized deep RNN.

In our work, first, features have been selected from the BCWD data set using correlation matrix. After that, two feature-selection algorithms, namely Univariate and RFE have been applied to features after correlation, and 11 features have been selected. Regular ML and the optimized deep RNN have been applied to the selected features, and the result of CV and the testing have been registered. Overall, the optimized deep RNN models have achieved the best performance for each feature-selection methods.  Figure 5 displays CV results of the optimized deep RNN results for each feature-selection methods. As can be seen, the deep RF has achieved the best performance using univariate (AC = 99.89%, PR = 99.89%, RE = 99.89%, and FM = 99.89%). Correlation and REF have recorded the same performance.  Figure 6 displays the testing results of the optimized deep RNN results for each feature-selection methods. As can be seen, the deep RNN has achieved the best performance using univariate (AC = 96.74%, PR = 96.39%, RE = 96.74%, and FM = 96.8%). Correlation and REF have recorded the same performance.

## 5. Conclusion

This paper used two approaches: the regular ML approach and the deep learning approach to predict breast cancer. In the DL approach, this paper proposes the optimized deep RNN model based on recurrent neural network (RNN) and the Keras–Tuner optimization technique. The optimized deep RNN consists of the input layer, six hidden layers, six dropout layers, and the output layer. In each hidden layer, we optimized the number of neurons and values of the dropout layer. In the regular ML approach, DT, RF, SVM, NB, and KNN were compared with the optimized deep RNN. Three feature-selection methods: correlation matrix, univariate, and REF were used to select the essential features from the database. The regular ML models and the optimized deep RNN are applied to selected features. The results show that the optimized deep RNN with selected features by univariate method has achieved the highest performance for cross-validation and testing results.

## Figures and Tables

**Table 1:** The breast cancer data set description.

SI. no.	Attribute	Attribute	Description
2	Diagnosis	Diagnosis	The identification of breast tissues (M = malignant, B = benign)
3	radius_mean	ra_m	Distances from the centre to the perimeter's points are averaged.
4	texture_mean	tex_m	Gray-scale value standard deviation
5	perimeter_mean	per_m	The tumor's average size
6	area_mean	ar_m	_
7	smoothness_mean	smo_m	Local difference in radius lengths mean
8	compactness_mean	com_m	Mean of perimeter2/area−1.0
9	concavity_mean	con_m	The average severity of the contour's concave parts
10	Concave points_mean	Con_po_m	The number of concave contour parts
11	symmetry_mean	sym_m	_
12	fractal_dimension_mean	fra_dim_m	Mean for “approximation of the shoreline”−1
13	radius_se	ra_s	Standard error for the mean of lengths from the centre to peripheral points
14	texture_se	te_s	Grayscale standard deviation standard error
15	perimeter_se	pe_s	_
16	area_se	ar_s	_
17	smoothness_se	smo_s	Standard error for local variation in radius lengths
18	compactness_se	com_s	Standard error for perimeter2/area−1.0
19	concavity_se	con_s	Standard error for the severity of the contour's concave parts
20	Concave points_se	Con_po_s	Number of concave parts of the contour standard error
21	symmetry_se	sym_s	_
22	fractal_dimension_se	fra_dim_s	Standard error for “a rough estimate of the coastline”−1
23	radius_worst	rad_w	“Worst” or largest mean value for the average of the distances between the centre and the points on the periphery
24	texture_worst	tex_w	“Worst” or largest mean value for gray-scale values' standard deviation
25	perimeter_worst	per_w	_
26	area_worst	ar_w	_
27	smoothness_worst	smo_w	“Worst” or largest mean value for variation in radius lengths on a local scale
28	compactness_worst	com_w	“Worst” or largest mean value for perimeter^2^/area−1.0
29	concavity_worst	con_w	“Worst” or largest mean value for the degree to which the contour is concave
30	Concave points_worst	Con_po_w	“Worst” or largest mean value for the contour's number of concave sections
31	symmetry_worst	sym_w	_
32	fractal_dimension_worst	fra_di_w	“Worst” or largest mean value for” approximation of the shoreline”–1

**Table 2:** The values of hyperparameters for the optimized deep RNN.

Hyperparameters	Values
Dropout rate	0.1–0.9
Number of neurons	50–700

**Table 3:** The performance of applying regular ML models and DL model with selected features by correlation matrix.

Approaches	Model	CV performance				Testing performance			
		AC	PR	RE	FM	AC	PR	RE	FM
Regular ML approach	DT	94.4	94.98	94.51	94.56	92.11	92.11	92.11	92.1
	KNN	89.85	90.63	89.85	89.51	86.67	87.4	86.67	86.17
	NB	81.84	82.38	81.84	81.01	83.68	84.33	83.68	83.0
	**RF**	**97.01**	**96.74**	**96.75**	**96.68**	**94.04**	**94.05**	**94.04**	**94.03**
	SVM	94.73	94.94	94.73	94.66	93.86	93.85	93.86	93.84

**DL approach**	**The optimized deep RNN**	**97.92**	**97.77**	**97.79**	**97.78**	**95.18**	**95.44**	**95.18**	**95.21**

**Table 4:** The number of neurons and dropout value in each layer in the optimized deep RNN for the selected features by correlation matrix.

Number of layers	Number of neurons in unit	Dropout layer
Input layer	190	0.8
Hidden layer1	470	0.4
Hidden layer2	90	0.7
Hidden layer3	630	0.4
Hidden layer4	370	0.4
Hidden layer5	270	0.4

**Table 5:** The scores of all features of applying univariate feature-selection method.

Feature	Score
ar_m	53 ,991.66
ar_s	8758.505
tex_m	93.897 51
con_w	39.516 92
con_m	19.712 35
sym_w	1.298 861
con_s	1.044 718
smo_w	0.397 366
sym_m	0.257 38
fra_di_wt	0.231 522
smo_m	0.149 899
tex_s	0.009 794
fra_dim_s	0.006 371
smo_s	0.003 266
sym_s	8.04E−05
fra_di_m	7.43E−05

**Table 6:** The performance of applying regular ML models and DL model with the selected features by univariate.

Approach	Model	CV performance				Testing performance			
		AC	PR	RE	FM	AC	PR	RE	FM
Regular ML approach	DT	95.17	95.11	94.73	94.72	89.04	89.32	89.04	89.1
	KNN	89.85	90.63	89.85	89.51	86.67	87.4	86.67	86.17
	**NB**	80.74	81.22	80.74	79.85	83.51	84.09	83.51	82.84
	**RF**	**96.57**	**96.52**	**96.44**	**96.41**	**94.00**	**94.00**	**94.00**	**94.00**
	SVM	93.85	94.07	93.85	93.78	93.86	93.85	93.86	93.84

**DL approach**	**The optimized deep RNN**	**99.89**	**99.89**	**99.89**	**99.89**	**96.74**	**96.39**	**96.74**	**96.8**

**Table 7:** The number of neurons and dropout value in each layer in the optimized deep RNN for the selected features by univariate.

Number of layers	Number of neurons in unit	Dropout layer
Input layer	550	0.3
Hidden layer1	230	0.9
Hidden layer2	390	0.3
Hidden layer3	490	0.9
Hidden layer4	170	0.9
Hidden layer5	330	0.4

**Table 8:** The performance of applying regular ML models and DL model with selected features by REF.

Approaches	Models	CV performance				Testing performance			
		AC	PR	RE	FM	AC	PR	RE	FM
Regular ML approach	DT	94.24	94.48	94.24	94.4	88.82	89.14	88.82	88.89
	KNN	89.85	90.63	89.85	89.51	86.67	87.4	86.67	86.17
	NB	80.74	81.22	80.74	79.85	83.51	84.09	83.51	**82.84**
	**RF**	**96.57**	**96.72**	**96.48**	**96.45**	**93.86**	**93.86**	**93.86**	**93.86**
	SVM	93.74	93.98	93.74	93.68	93.86	93.85	93.86	93.84

DL approach	**The optimized deep RNN**	**97.92**	**97.77**	**97.79**	**97.78**	**95.18**	**95.44**	**95.18**	**95.21**

**Table 9:** The number of neurons and dropout value in each layer in the optimized deep RNN for the selected features by REF.

Number of layers	Number of neurons in unit	Dropout layer
Input layer	190	0.8
Hidden layer1	470	0.4
Hidden layer2	90	0.7
Hidden layer3	630	0.4
Hidden layer4	370	0.5
Hidden layer5	270	0.4

## Data Availability

Breast Cancer Wisconsin (diagnostic) data set can be downloaded from https://www.kaggle.com/uciml/breast-cancer-wisconsin-data, 2021.
